# Prevalence of falls, injury from falls and associations with chronic diseases among community-dwelling older adults in Guangzhou, China: a cross-sectional study

**DOI:** 10.3389/fpubh.2023.1251858

**Published:** 2023-09-15

**Authors:** Wei-Quan Lin, Lin Lin, Si-Yu Sun, Le-Xin Yuan, Min-Ying Sun, Chang Wang, Jia-Min Chen, Yao-Hui Li, Qin Zhou, Di Wu, Ting-Yuan Huang, Bo-Heng Liang, Hui Liu

**Affiliations:** ^1^Department of Basic Public Health, Center for Disease Control and Prevention of Guangzhou, Guangzhou, China; ^2^Institute of Public Health, Guangzhou Medical University and Guangzhou Center for Disease Control and Prevention, Guangzhou, China; ^3^School of Public Health, Guangzhou Medical University, Guangzhou, China; ^4^School of Public Health, Southern Medical University, Guangzhou, China; ^5^Brain Hospital of Guangzhou Medical University, Guangzhou Huiai Hospital, Guangzhou, China; ^6^Department of Prevention and Control of Chronic Noncommunicable Diseases, Center for Disease Control and Prevention of Guangzhou, Guangzhou, China

**Keywords:** falls, injury from falls, older adults, risk factors, chronic diseases

## Abstract

**Introduction:**

As a developing country with the largest older adult population in the world, strengthening the research on falls among the older adults is undoubtedly an urgent item in China. This study aimed to explore the prevalence and risk factors associated with falls and injury from falls among community-dwelling older adults in Guangzhou, China, particularly focusing on their associations with chronic diseases.

**Methods:**

A total of 1,629 participants aged 65 years and above were selected from 11 counties in Guangzhou by the multi-stage stratified random sampling method in 2021. Socio-demographic characteristics, health and lifestyle factors, the status of falls, and injury from falls were measured by structured questionnaires through face-to-face interviews. Chi-square tests and logistic regression analysis were used to identify factors associated with falls and injury from falls. Chord diagrams were used to explore their associations with chronic diseases.

**Results:**

A total of 251 participants (15.41%, 95% CI: 13.98%−17.25%) reported falls, and 162 participants (9.46%, 95% CI:7.72%−11.55%) indicated an injury from falls. Logistic regression analysis showed the results as follows: female patients (adjusted OR = 1.721, 95% CI: 1.681–1.761) aged ≥80 years (1.910, 1.847–1.975), unemployed (1.226, 1.171–1.284), uninsured (1.555, 1.448–1.671), average monthly household income of 2,001–4,000 CNY (1.878, 1.827–1.930), number of services provided by the community health center ≥13 times per year (1.428, 1.383–1.475), illness within 2 weeks (1.633, 1.595–1.672), high-intensity physical activity (2.254, 2.191–2.32), sedentary (1.094, 1.070–1.117), and number of chronic disease illnesses ≥3 (1.930, 1.870–1.993). Meanwhile, those risk factors were also associated with injury from falls. The older adults with medium-intensity physical activity were at lower risk (0.721, 0.705–0.737) of falls and higher risk (1.086, 1.057–1.117) of being injured from falls. Chord diagrams showed the correlations between chronic diseases and falls and injury from falls among community-dwelling older adults in Guangzhou, China.

**Conclusion:**

The high prevalence of falls is found among community-dwelling older adults in Guangzhou, China, which is related to multiple factors such as demographic variables, lifestyle, and health status, especially for chronic diseases. Therefore, targeted interventions should be developed and implemented urgently.

## Introduction

Data from the National Bureau of Statistics in China showed that individuals aged 65 years or above constitute ~2.10 million (14.9%) of the total population in 2022 (1). China is experiencing a rapidly growing aging population. Falls are defined as an unexpected, unintentional change in position that causes an individual to remain at a lower level (2). Falls not only damage the health and quality of life but also bring a heavy burden to families and society. A previous study reported that falls are the second leading cause of unintentional injury deaths worldwide (3). Each year an estimated 684,000 individuals die from falls globally of which over 80% are in low- and middle-income countries (3). Moreover, falls are the number one cause of injury-related death among people aged 65 years and above (4). Based on the Guangzhou Injury Monitoring System, injury with falls occupied the first place from 2014 to 2018 (5). Falls of older adults have become a growing public health problem in China. Therefore, under the background of the construction of healthy China, strengthening the research on falls among older adults is undoubtedly an urgent item in China.

Approximately 30% of community-dwelling older adults fall each year, and 5%−10% of falls result in serious injuries, such as head injuries or fractures (6). Previous studies have shown that the prevalence of falls in older people varied between 8.59 and 39.7% (7–10). In China, the prevalence of falls among community-dwelling older adults is 22.49%, and the injury from falls rate is 8.00% (11). The wide variations in fall prevalence in those studies may be related to the definition of falls, the assessment methods, and racial differences. All of these studies indicate that the prevalence of falls remains high and that it has become a serious public health problem deserving more attention.

Risk factors for the occurrence of falls in older adults are complex. Falls are usually caused by a combination of risk factors. Gender differences in the prevalence of falls exist (10, 12, 13). With age, the risk of falling increases for people aged 60 years and above (14). In addition, having a chronic disease is also associated with the risk of injury from falls, including many chronic conditions such as diabetes (15), hypertension (4), and cardiovascular disease (16). A Canadian community health survey showed that the higher the number of chronic diseases in middle-aged and older adults, the higher the risk of injury from falls (4). However, a limited study was reported on associations between falls, injury from falls, and chronic diseases among community-dwelling older adults in Guangzhou, China.

Therefore, the study aimed to explore the prevalence and risk factors associated with falls and injury from falls among community-dwelling older adults in Guangzhou, China, particularly focusing on their associations with chronic diseases.

## Methods

### Study design and sampling

This was a cross-sectional study about the prevalence of falls and injury from falls among 1,629 participants aged more than 65 years and their relationship with risk factors in 2021 in Guangzhou. Structured questionnaires through face-to-face interviews were adopted, and the interviews were conducted by a group composed of local healthcare staff and medical students in the present study.

**Figure 1:**
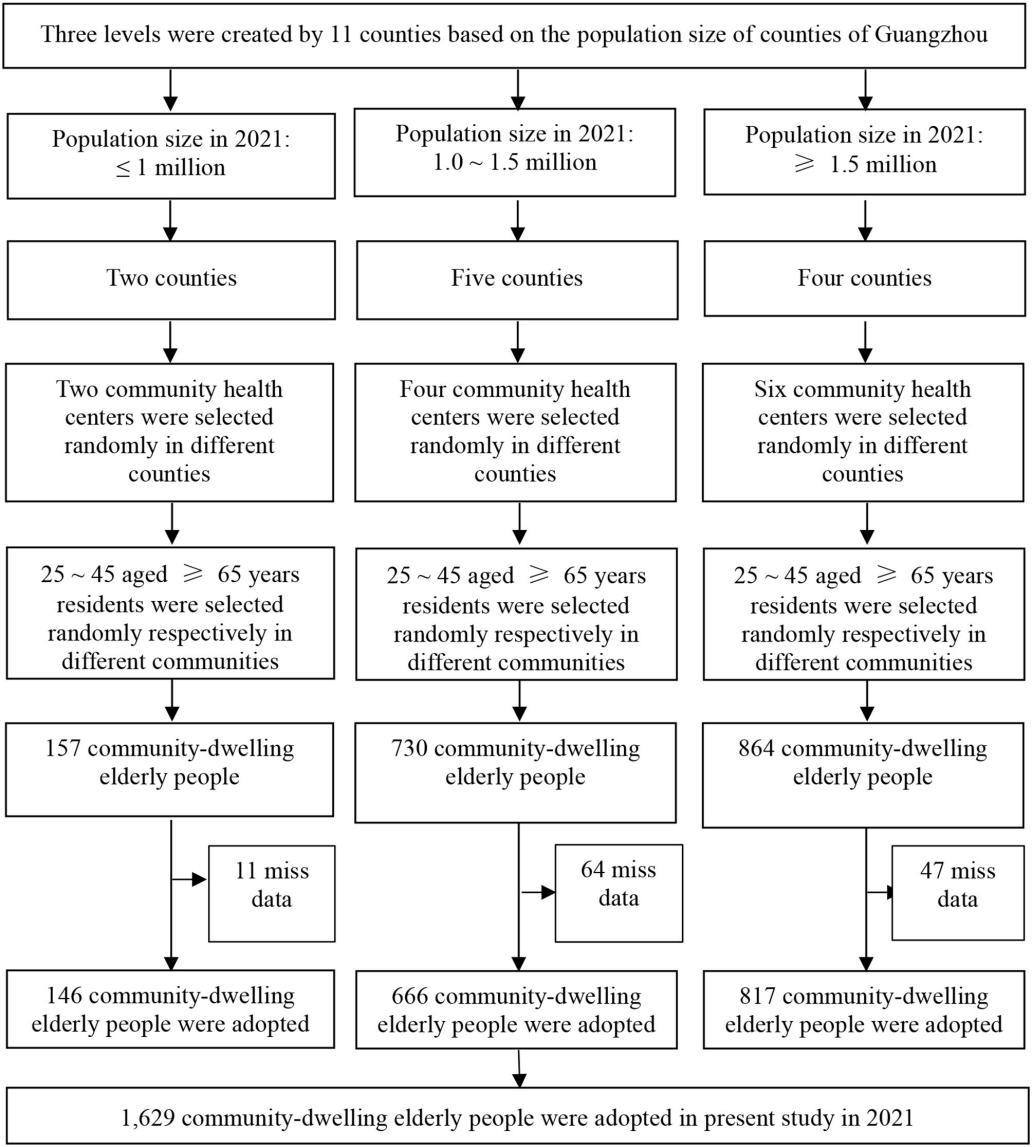
Sampling framework in this study in Guangzhou, China.

Participants were selected based on a multi-step stratified random sampling method. Initially, the 11 counties were divided into three classes in accordance with the total population of each district, and the same number of community health centers was randomly chosen in each class according to the distribution of community centers for the study. That is to say, two community centers were selected from two districts with a population of <1 million; four community centers were selected from five areas with a population of 1–1.5 million; and six community centers were selected from four regions with a population >1.5 million. Subsequently, 25–45 residents aged 65 years were randomly selected at each community center, with a total of 1,751 seniors selected. Following data processing, 122 individuals with missing information were excluded, and a total of 1,629 patients were included. The sampling framework is displayed in Figure 1 in this study.

### Instruments

A self-designed questionnaire of the health status of older people was adopted in the present study, which was constituted by socio-demographic characteristics, health and lifestyle factors, and assessment of falls, injury from falls (Supplementary material 1).

### Socio-demographic characteristics

These measured the socio-demographic characteristics, including gender, age groups, education level, occupations, marital status, medical insurance, registered residence in Guangzhou, and monthly household income per head.

### Health and lifestyle factors

The health and lifestyle factors section included health-related factors and lifestyle-related factors. Health-related factors included chronic diseases (hypertension, diabetes, coronary heart disease, chronic respiratory disease, stroke, renal disease, hyperlipidemia, hepatic disease, high uric acid, tumor, and other chronic diseases), number of chronic diseases, number of services provided by community health service center per year, 2-week prevalence rate, and health status. Lifestyle-related factors included whether they engaged in low, medium, and high levels of physical activity and whether they had sedentary behavior. Low-intensity physical activity was defined as older people having engaged in low-intensity physical activities for 10 min or more every day, such as taking a walk, in the past week. Medium-intensity physical activity was defined as older people having engaged in medium-intensity physical activities for 10 min or more at least 3 days of the week, such as lifting light objects or playing Tai Chi, in the past week. High-intensity physical activity was defined as older people having engaged in high-intensity physical activities for 10 min or more in the past week, such as lifting heavy objects and playing basketball. Sedentary behavior was defined as older people having accumulated sitting time ≥4 h within a day.

### Assessment of falls, injury from falls

The assessment of falls was based on a question: Have you fallen in the past year? The assessment of injury from falls was based on a question: Have you been injured from falls in the past year?

### Ethics statement

Ethical approval for this study was obtained from the Ethics Committee of the Center for Disease Control and Prevention of Guangzhou.

### Statistical analyses

Statistical analyses were performed using R 4.0.0 and SPSS 21.0 (SPSS Inc., Chicago, IL). Differences between dependent variables and falls and falls with injury among older adults were examined using chi-square tests. Binary logistic regression analysis was used to identify related factors associated with falls and injury from falls. The independent variables were explored using the maximum likelihood method. The odds ratio (OR) and 95% confidence interval (CI) were reported. Chord diagrams were used to explore the association with chronic disease. To increase the representativeness of the study population, all statistics were calculated by using base weights adjustment (population weight and post-stratification sample weights). A *p*-value of <0.05 was considered significant in all performed analyses.

## Result

A total of 1,629 older people aged 65 years or above were surveyed in this study from Guangzhou, 62.31% were female, 88.65% were educated, 57.64% were workers, 84.53% were married, 98.92% had medical insurance, and 87.85% were registered in Guangzhou (Table 1).

**Table 1:** Prevalence of falls and injury from falls and its association with socio-demographic characteristics among community-dwelling older people in Guangzhou, China.

**Variables**	**Total**		**Prevalence of falls**		***P*-value^c^**	**Prevalence of injury from falls**		***P*-value^c^**
	***n***	**%**	***n***	**% (95% CI)** ^b^		***n***	**% (95% CI)** ^b^	
**Gender**					< 0.001			< 0.001
Male	614	37.69	73	12.12 (10.31–14.20)		36	5.46 (3.77–7.84)	
Female	1,015	62.31	178	18.64 (15.57–22.16)		126	13.08 (10.35–16.41)	
**Age groups (years)**					< 0.001			< 0.001
65–69	681	41.80	91	12.81 (10.43–15.62)		61	7.89 (6.20–9.99)	
70–74	505	31.00	78	15.01 (11.15–19.91)		44	8.21 (5.92–11.28)	
75–79	280	17.19	46	16.66 (12.17–22.39)		32	10.34 (5.43–18.80)	
≥80	163	10.01	36	23.00 (17.40–29.73)		25	15.12 (11.21–20.09)	
**Education level**					< 0.001			< 0.001
No school	185	11.36	34	16.50 (8.31–30.09)		23	11.46 (5.50–22.35)	
Primary school	595	36.53	89	15.91 (12.43–20.12)		55	9.03 (6.56–12.30)	
Secondary school	699	42.91	103	15.25 (12.65–18.26)		69	9.59 (6.51–13.91)	
College and above	150	9.21	25	14.36 (10.31–19.66)		15	7.92 (5.16–11.95)	
**Occupations**					< 0.001			< 0.001
Worker	939	57.64	139	14.79 (12.29–17.7)		95	9.92 (7.71–12.69)	
Technical personnel	235	14.43	50	22.97 (19.59–26.73)		31	13.23 (8.71–19.59)	
Unemployed	223	13.69	29	14.27 (10.19–19.61)		13	5.72 (3.60–8.96)	
Others	232	14.24	33	13.08 (9.34–18.02)		23	7.44 (3.97–13.52)	
**Marital status**					< 0.001			< 0.001
Single^a^	252	15.47	46	19.02 (12.76–27.39)		30	11.28 (6.23–19.56)	
Married	1,377	84.53	205	14.86 (13.26–16.63)		132	9.11 (7.74–10.70)	
**Medical insurance**					< 0.001			0.061
Uninsured	28	1.72	6	20.76 (11.06–35.55)		2	10.20 (2.84–30.63)	
Insured	1,601	98.28	245	15.45 (13.9–17.13)		160	9.45 (7.72–11.51)	
**Registered residence in Guangzhou**					< 0.001			< 0.001
Yes	1,431	87.85	216	15.14 (13.56–16.88)		142	9.22 (7.23–11.70)	
No	198	12.15	35	18.54 (13.78–24.48)		20	11.26 (6.94–17.77)	
**Monthly household income per head (**¥**, RMB)**					< 0.001			< 0.001
≤ 2,000	477	29.28	71	13.17 (9.52–17.94)		40	7.01 (4.31–11.21)	
2,001–4,000	460	28.24	91	20.68 (17.51–24.27)		63	13.48 (10.37–17.33)	
4,001–6,000	413	25.35	62	15.31 (12.33–18.87)		42	10.05 (7.19–13.88)	
>6,000	279	17.13	27	11.46 (7.67–16.78)		17	6.39 (3.79–10.59)	
All participants	1,629	100.00	251	15.55 (13.98–17.25)		162	9.46 (7.72–11.55)	

a Single: unmarried, divorced, or widowed.

b Weighted estimates of prevalence of with proportional to population size and post-stratification sample weights adjustment.

c Differences between means within each variable, chi-square test analysis for independent samples.

**Figure 2:**
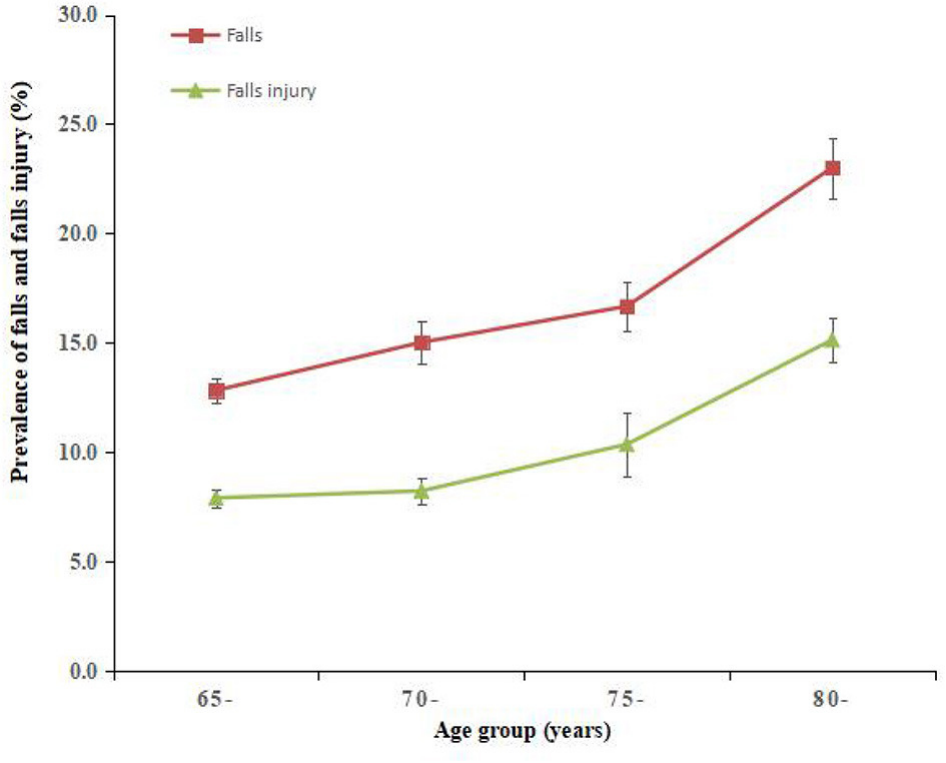
Prevalence of falls and injury from falls by age group among community-dwelling older adults in Guangzhou, China. Error bars indicate standard error.

**Figure 3:**
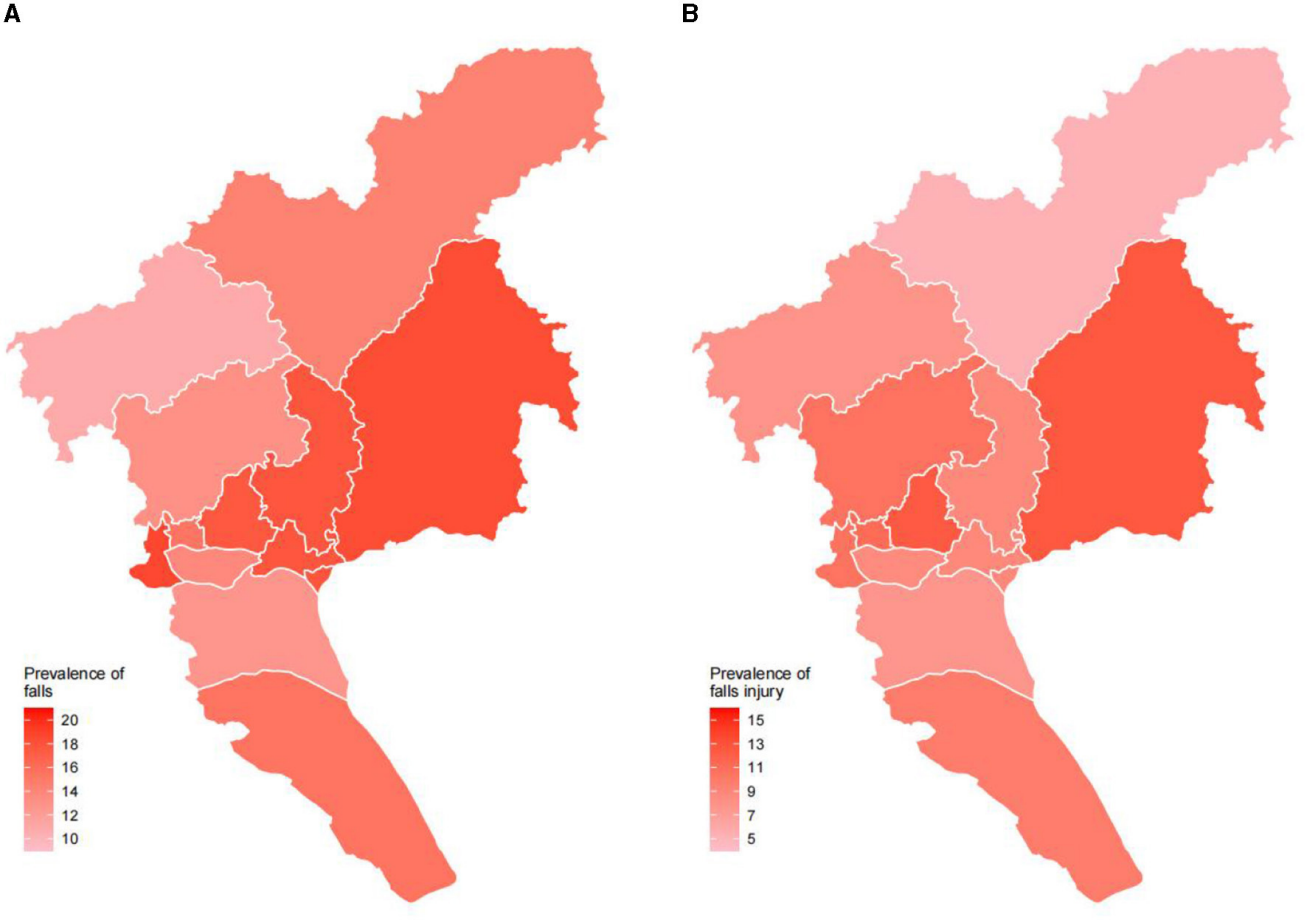
Prevalence of falls and injury from falls by region among community-dwelling older people in Guangzhou, China. **(A)** Prevalence of falls. **(B)** Prevalence of injury from falls.

In the comparison of the study population and the seventh national population census of Guangzhou in 2020, differences were found between age groups and gender for the chi-square test analysis (Supplementary Table S1). To estimate the prevalence of falls and injury from falls, population weight and post-stratification sample weight adjustment were adopted in this study. A total of 251 (15.41%, 95% CI: 13.98%−17.25%) participants reported falls, and 162 (9.46%, 95% CI: 7.72%−11.55%) participants indicated an injury from falls. The chi-square test in Table 1 shows the demographic factors, age, gender, education level, occupation, marital status, residence, and monthly household income per head were associated with the occurrence of both falls and injury from falls, while medical insurance was only associated with falls. The prevalence of falls and injury from falls increased with age group among participants (Figure 2). Figure 3 shows the prevalence of falls and injury from falls by region participants.

Among lifestyle factors, we did not observe that low physical activity was associated with fall risk, but high and moderate physical activity and sedentary time were all associated with falls, while injury from falls was associated with all lifestyle factors in the chi-square test (Table 2). The results of the study demonstrated that hypertension, diabetes, coronary heart disease, chronic respiratory disease, stroke, hepatic disease, tumors, and the number of chronic diseases suffered were all related to falls and injury from falls in older adults, while kidney disease and hyperlipidemia were only associated with falls (Table 2).

**Table 2:** Prevalence of falls and injury from falls and its association with health and lifestyle factors among community-dwelling older people in Guangzhou, China.

**Variables**	**Total**		**Prevalence of falls**		***P*-value^b^**	**Prevalence of injury from falls**		***P*-value^b^**
	***n***	**%**	***n***	**% (95% CI)** ^a^		***n***	**% (95% CI)** ^a^	
**Health-related factors**								
**Hypertension**					< 0.001			< 0.001
Yes	1006	61.76	157	15.86 (13.51–18.52)		106	9.78 (7.85–12.12)	
No	623	38.24	94	15.02 (12.65–17.74)		56	8.94 (5.88–13.35)	
**Diabetes**					< 0.001			< 0.001
Yes	565	34.68	112	20.34 (16.96–24.21)		66	10.87 (7.87–14.83)	
No	1,064	65.32	139	12.87 (11.11–14.86)		96	8.68 (6.86–10.93)	
**Coronary heart disease**					< 0.001			< 0.001
Yes	209	12.83	41	21.53 (14.64–30.50)		31	14.32 (11.07–18.31)	
No	1,420	87.17	210	14.66 (12.70–16.85)		131	8.75 (7.02–10.85)	
**Chronic respiratory disease**					0.031			< 0.001
Yes	52	3.19	12	16.35 (6.52–35.36)		11	14.95 (6.09–32.26)	
No	1,577	96.81	239	15.52 (13.87–17.32)		151	9.28 (7.62–11.27)	
**Other chronic diseases**					0.003			< 0.001
Yes	49	3.01	9	14.36 (6.35–29.32)		7	11.51 (4.62–25.86)	
No	1,580	96.99	242	15.58 (13.90–17.42)		155	9.41 (7.72–11.42)	
**Stroke**					< 0.001			< 0.001
Yes	46	2.82	16	38.24 (17.68–64.09)		10	23.02 (9.82–45.10)	
No	1,583	97.18	235	14.86 (13.35–16.50)		152	9.05 (7.14–11.41)	
**Renal disease**					< 0.001			0.998
Yes	42	2.58	11	28.67 (16.17–45.58)		5	9.47 (2.73–28.08)	
No	1,587	97.42	240	15.18 (13.38–17.16)		157	9.46 (7.66–11.64)	
**Hyperlipidemia**					0.010			0.158
Yes	37	2.27	5	14.25 (5.61–31.73)		3	8.90 (1.90–33.02)	
No	1,592	97.73	246	15.57 (14.04–17.23)		159	9.48 (7.64–11.69)	
**Hepatic disease**					< 0.001			< 0.001
Yes	33	2.03	9	24.98 (6.06–63.22)		7	14.92 (3.43–46.41)	
No	1,596	97.97	242	15.3 (13.46–17.35)		155	9.32 (7.32–11.81)	
**High uric acid**					< 0.001			< 0.001
Yes	25	1.53	2	4.28 (0.85–18.89)		2	4.28 (0.85–18.89)	
No	1,604	98.47	249	15.77 (14.25–17.42)		160	9.57 (7.84–11.63)	
**Tumor**					< 0.001			< 0.001
Yes	8	0.49	3	41.05 (10.47–80.57)		2	15.09 (4.09–42.53)	
No	1,621	99.51	248	15.40 (13.80–17.16)		160	9.43 (7.70–11.52)	
**Number of chronic disease**					< 0.001			< 0.001
0–2	1,481	90.91	209	14.04 (11.89–16.52)		132	8.53 (6.48–11.15)	
≥3	148	9.09	42	29.43 (17.66–44.78)		30	18.10 (10.81–28.71)	
**Number of services provided by community health service**								
**center per year**					< 0.001			< 0.001
0–2	298	18.29	37	13.13 (7.76–21.37)		24	9.03 (4.39–17.67)	
3–12	986	60.53	141	14.79 (11.56–18.74)		92	8.80 (6.83–11.28)	
≥13	345	21.18	73	20.03 (13.91–27.97)		46	11.7 (8.09–16.62)	
**Two-week prevalence rate**					< 0.001			< 0.001
Yes	358	21.98	87	23.13 (20.08–26.50)		58	14.74 (11.07–19.36)	
No	1,271	78.02	164	13.12 (11.07–15.48)		104	7.78 (6.02–10.00)	
**Health status**					< 0.001			< 0.001
Better than contemporary	376	23.08	48	13.10 (9.86–17.2)		29	8.21 (6.30–10.63)	
Same as contemporary	976	59.91	137	13.70 (11.29–16.52)		85	8.21 (6.10–10.95)	
Worse than contemporary	277	17.00	66	23.93 (20.00–28.35)		48	14.68 (10.90–19.49)	
**Lifestyle-related factors**								
**High-intensity physical activity**					< 0.001			< 0.001
Yes	215	13.20	45	20.49 (15.07–27.22)		24	8.17 (3.67–17.21)	
No	1,414	86.80	206	14.73 (13.01–16.63)		138	9.68 (8.22–11.36)	
**Medium-intensity physical activity**					< 0.001			< 0.001
Yes	1,031	63.29	154	13.98 (10.90–17.76)		104	9.72 (7.98–11.78)	
No	598	36.71	97	18.11 (15.02–21.67)		58	9.05 (7.14–11.40)	
**Low intensity physical activity**					0.790			< 0.001
Yes	1,474	90.48	227	15.54 (13.70–17.58)		146	9.34 (7.56–11.50)	
No	155	9.52	24	15.59 (10.86–21.90)		16	10.42 (6.49–16.30)	
**Sedentary behavior (hours)**					< 0.001			< 0.001
≥4	591	36.28	106	16.07 (11.55–21.91)		68	10.29 (8.17–12.88)	
< 4	1,038	63.72	145	15.17 (12.93–17.73)		94	8.88 (6.27–12.42)	
All participants	1,629	100.00	251	15.55 (13.98–17.25)		162	9.46 (7.72–11.55)	

a Weighted estimates of prevalence of with proportional to population size and post-stratification sample weights adjustment.

b Differences between means within each variable, chi-square test analysis for independent samples.

**Figure 4:**
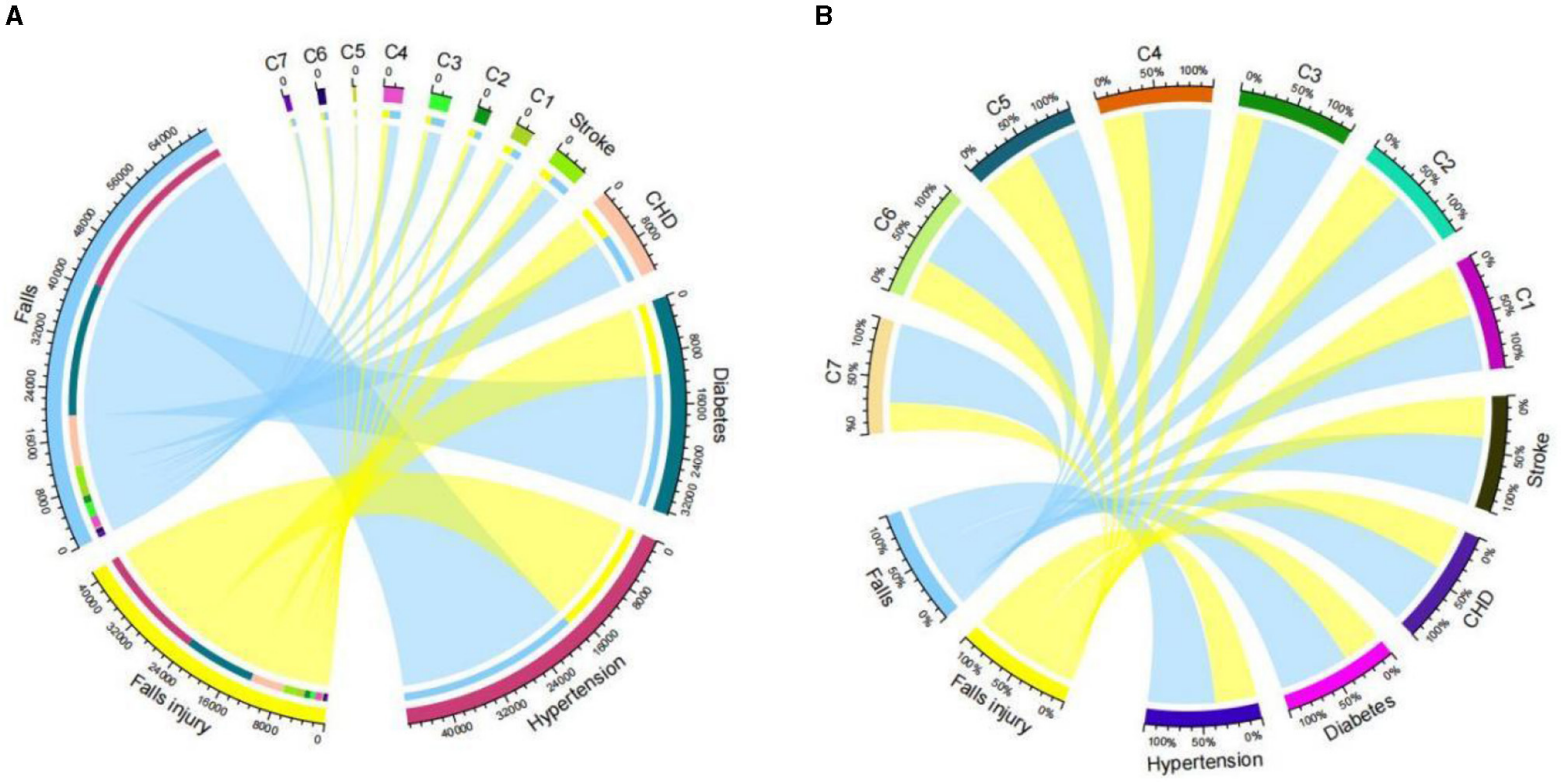
Chord diagrams about falls and injury from falls associated with chronic disease among community-dwelling older people in Guangzhou, China. **(A)** Number of falls and injury from falls. **(B)** % of falls and injury from falls. CHD, coronary heart disease; C1, chronic respiratory disease; C2, other chronic diseases; C3, renal disease; C4, hepatic disease; C5, high uric acid; C6, hyperlipidemia; C7, tumor.

Logistic regression analysis (Table 3) showed that the risk factors for falls in older adults were: female [adjusted OR = 1.721, 95% CI (1.681–1.761)], aged ≥80 years [OR = 1.910, 95% CI (1.847–1.975)], unemployed [OR = 1.226, 95% CI (1.171–1.284)], uninsured [OR = 1.555, 95% CI (1.448–1.671)], average monthly household income of 2,001–4,000 CNY [OR = 1.878, 95% CI (1.827–1.930)], number of services provided by the community health center ≥13 times per year [OR = 1.428, 95% CI (1.383–1.475)], illness within 2 weeks [OR = 1.633, 95% CI (1.595–1.672)], high-intensity physical activity [OR = 2.254, 95% CI (2.191–2.32)], sedentary [OR = 1.094, 95% CI (1.070–1.117)], and number of chronic disease illnesses ≥3 [OR = 1.930, 95% CI (1.870–1.993)]. Meanwhile, those risk factors were also associated with injury from falls. The older adults with medium-intensity physical activity were at lower risk [OR = 0.721, 95% CI (0.705–0.737)] of falling and at higher risk [OR = 1.086, 95% CI (1.057–1.117)] of injury from falls. Chord diagrams (Figure 4) showed the correlations between chronic diseases and falls and injury from falls among community-dwelling older adults in Guangzhou, China.

**Table 3:** Association of falls and injury from falls with socio-demographic and health and lifestyle factors among community-dwelling older people in Guangzhou, China.

**Variables**	**Falls**		**Injury from falls**	
	**OR (95% CI)** ^a^	***P*** **-value** ^b^	**OR (95% CI)** ^a^	***P*** **-value** ^b^
**Socio-demographic characteristics**				
**Gender**				
Male	Reference	Reference
Female	1.721 (1.681–1.761)	< 0.001	2.630 (2.551–2.710)	< 0.001
**Age groups (years)**				
65–69	Reference	Reference
70–74	1.260 (1.224–1.296)	< 0.001	1.123 (1.083–1.164)	< 0.001
75–79	1.182 (1.149–1.216)	< 0.001	1.183 (1.143–1.224)	< 0.001
≥80	1.910 (1.847–1.975)	< 0.001	1.917 (1.841–1.996)	< 0.001
**Education level**				
No school	Reference	Reference
Primary school	1.009 (0.974–1.046)	0.614	0.878 (0.842–0.916)	< 0.001
Secondary school	1.113 (1.072–1.156)	< 0.001	1.210 (1.157–1.265)	< 0.001
College and above	1.034 (0.979–1.092)	0.228	1.110 (1.038–1.188)	0.002
**Occupations**				
Others	Reference	Reference
Worker	1.760 (1.692–1.831)	< 0.001	1.533 (1.459–1.611)	< 0.001
Technical personnel	1.000 (0.965–1.036)	0.997	1.221 (1.168–1.277)	< 0.001
Unemployed	1.226 (1.171–1.284)	< 0.001	0.843 (0.792–0.898)	< 0.001
**Medical insurance**				
Insured	Reference	Reference
Uninsured	1.555 (1.448–1.671)	< 0.001	1.104 (1.005–1.212)	0.039
**Registered residence in Guangzhou**				
No	Reference	Reference
Yes	0.686 (0.664–0.708)	< 0.001	0.775 (0.745–0.805)	< 0.001
**Monthly household income per head (**¥**, RMB)**				
≤ 2,000	Reference	Reference
2,001–4,000	1.878 (1.827–1.930)	< 0.001	2.020 (1.952–2.091)	< 0.001
4,001–6,000	1.138 (1.102–1.174)	< 0.001	1.335 (1.284–1.388)	< 0.001
>6,000	0.775 (0.744–0.808)	< 0.001	0.839 (0.796–0.884)	< 0.001
**Health-related factors**				
**Number of chronic disease**				
0–2	Reference	Reference
≥3	1.930 (1.870–1.993)	< 0.001	1.619 (1.559–1.682)	< 0.001
**Number of services provided by community health service center per year**				
0–2	Reference	Reference
3–12	1.095 (1.065–1.127)	< 0.001	0.937 (0.906–0.969)	< 0.001
≥13	1.428 (1.383–1.475)	< 0.001	1.103 (1.060–1.146)	< 0.001
**Two-week prevalence rate**				
No	Reference	Reference
Yes	1.633 (1.595–1.672)	< 0.001	1.580 (1.536–1.626)	< 0.001
**Health status**				
Worse than contemporary	Reference	Reference
Same as contemporary	0.578 (0.559–0.597)	< 0.001	0.594 (0.571–0.618)	< 0.001
Better than contemporary	0.572 (0.556–0.587)	< 0.001	0.577 (0.558–0.596)	< 0.001
**Lifestyle-related factors**				
**High-intensity physical activity**				
No	Reference	Reference
Yes	2.254 (2.191–2.32)	< 0.001	1.260 (1.211–1.311)	< 0.001
**Medium-intensity physical activity**				
No	Reference	Reference
Yes	0.721 (0.705–0.737)	< 0.001	1.086 (1.057–1.117)	< 0.001
**Sedentary behavior (years)**				
< 4	Reference	Reference
≥4	1.094 (1.070–1.117)	< 0.001	1.219 (1.188–1.252)	< 0.001

a Adjusted for all variables listed in the table. OR, odds ratio; CI, confidence interval.

b P-value for OR.

## Discussion

Our study found that the prevalence of falls was 15.41% among community-dwelling older adults in Guangzhou, China, which was similar to those found in most East Asian regions, such as Singapore, Korea, Japan, and some areas (Taiwan and Chengdu City) of China (17–22). A systematic review showed that the prevalence of falls among older people in China was 0.6%−19.5% (23). However, it was lower than Brazil (2), Nigeria (24), the USA (25), Canada (26), and France (27), in which all reported rates were closer to a fifth of their studied population. A systematic review of the world's older people showed that the prevalence of falls was 26.5% (28). The differences between these regions may be related to different racial, economic, and culturally related factors that may play a role in falls among older adults. Our study found that the prevalence of injury from falls was 9.46% among community-dwelling older adults in Guangzhou, China, which was close to the previous study (29, 30). It was higher than some previous studies that reported in China: 5.02%, 6.78% in Beijing (31, 32), 4.09% in Shijiazhuang (30), 3.85% in Taiwan (33), and 2.69% in Shanghai (34). This may be related to the large number of older people in Guangzhou and the increasing aging of the population. Moreover, differences in the definition of falls and the choice of study subjects and methods may help explain this phenomenon. Nonetheless, falls and injury from falls were urgent needs for the exploration of risk factors and implementation of interventions among community-dwelling older people in Guangzhou, China.

In the present study, the prevalence of falls increased with the age of the respondents, consistent with other studies (31, 35). It may be because that postural control, body orientation reflexes, muscle strength and tone, and stride height decrease with age in older adults and the ability to avoid falls. Similar to our result, numerous studies have reported that women are more vulnerable to falls and injury from falls than men (OR = 1.721) (36). Women are more likely to suffer from osteoporosis owing to the lack of estrogen after menopause; and as they get aged, women's lower limb muscle strength declines faster than men of the same age. This suggests that we should strengthen the prevention and treatment of osteoporosis in older adults.

Some experiments suggested that college or university education was a protective factor against falls (10). The results of this experiment showed that educational background was a protective factor against falls similar to previous research studies in Africa and China (37, 38). Other studies have suggested that education is not associated with falls (39). The prevalence of falls and injuries of people registered in Guangzhou is less than in others. The reason may be that Guangzhou is a city for migrant workers. Most of the older people registered in Guangzhou have retired and do not need to go to work, which reduces the risk of falling.

Compared with older families with higher incomes, a higher risk of prevalence of falls and injury from falls was found among low-income older families. Meanwhile, our study demonstrated that older people without insurance had a higher risk of falling than those who possessed coverage. The absence of insurance may be associated with low income. Low income may be associated with poor living conditions, poor health behaviors, and barriers to health services, which may affect health status and increase the risk of falls. Furthermore, uninsured older people may not have regular healthcare to maintain good health, strength, or health literacy to prevent falls. Working status and education level are important indicators of socioeconomic status, and studies have shown that older people with low socioeconomic status are more likely to fall (40). There was also literature suggesting that the overall picture was more complicated (40, 41).

In the present study, older adults with high physical activity had a higher risk of falls and injury from falls, and those with moderate physical activity had a lower risk of prevalence of falls while had a higher risk of prevalence injury of falls interestingly. For the decreased muscle strength and balance ability, suddenly doing high physical activity, such as lifting heavy objects or playing basketball, may not be suitable for older people. Meanwhile, sitting for long periods is a risk factor for falls and injury from falls. Sedentary behavior increases the risk of the prevalence of diseases such as type 2 diabetes, cardiovascular disease, and hypertension (42). In addition, sedentary behavior is negatively correlated with bone mineral density (42), which may increase the risk of falls and injury from falls. Therefore, it should be recommended that older people exercise appropriately according to their physical strength to improve their balance and muscle strength. Tai chi, as a traditional physical exercise program, has been proven to be effective in preventing falls among older adults in the community (43, 44), which may reduce the rate of falls and injury-related falls over the short term by ~43 and 50% (44).

Our study found that falls and injury from falls were associated with their health conditions. Many studies showed that falls, both with and without injuries are associated with the subsequent decline in physical function (45). The psychological health of older adults may contribute to this phenomenon. Studies have shown that people who fear falling for various reasons are more likely to fall (46). A 5-year community-dwellings' fall prevention health education intervention project in Australia showed that the awareness rate of fall prevention increased by 17% and ultimately reduced the incidence of falls by 22% (47). Therefore, community health centers can carry out health education to eliminate the fear of older adults, increase their awareness of fall health knowledge, and ultimately achieve the purpose of fall prevention.

A study of 16,357 community-dwelling seniors in Canada showed that people without chronic conditions had a much lower risk of falls than people with any chronic condition; the risk of falls increased with the number of chronic conditions (4). Our findings further showed that older people with illness in the last 2 weeks and more chronic diseases were the risk factors for falls and injury from falls. Notably, older people with three or more chronic conditions reported significantly more often having been injured by falling.

Hypertension and diabetes are common chronic diseases among older people. Our study found that older people with hypertension had a higher prevalence of falls and injury with falls than those without hypertension possibly as a side effect of treatment with medication that can trigger orthostatic hypotension. In a case–control study, older people in the UK who received thiazide drugs were associated with an increased risk of falling, and this risk was greatest within 3 weeks of the first prescription (48). Equally, those with diabetes had a higher prevalence of falls than those without diabetes, which was consistent with a previous study (15). It has been shown that the occurrence of hypoglycemia may contribute to falls in older diabetic patients and the prevalence of falls increases with the frequency of hypoglycemia (49). It may be due to cognitive impairments that exist during hypoglycemia, such as lack of concentration and slowed psychomotor speed, and will persist for 30 min after recovery from hypoglycemia, leading to a fall (50).

The strength of this study is its population-based sample and analysis. However, some limitations of this study need to be considered. First, due to the cross-sectional nature of this study design, causal relationships could not be firmly deduced. Second, though face-to-face interviews were adopted in the present study, recall bias might be introduced. Third, some possible risk factors were neglected, such as the history of falls and depression, and the definitions of falls and injury from falls were only based on the response to individual perspectives question. Therefore, the causal relationships in this study require larger and more prospective cohort studies to confirm them.

## Conclusion

The high prevalence of falls is found among community-dwelling older people in Guangzhou, China, which is related to multiple factors such as demographic variables, lifestyle, and health status, especially for chronic diseases. Therefore, targeted interventions should be developed and implemented urgently.

## Data availability statement

The raw data supporting the conclusions of this article will be made available by the authors, without undue reservation.

## Ethics statement

Ethical approval for this study was obtained from the Ethics Committee of the Center for Disease Control and Prevention of Guangzhou. The studies were conducted in accordance with the local legislation and institutional requirements. The participants provided their written informed consent to participate in this study.

## Author contributions

W-QL, T-YH, M-YS, CW, J-MC, Y-HL, QZ, DW, and B-HL supervised the study data collection and quality control. W-QL and S-YS conducted the literature review. W-QL and M-YS conducted the data analyses. W-QL, LL, and S-YS drafted the manuscript. W-QL and HL finalized the manuscript with inputs from all authors. All authors contributed to the article and approved the submitted version.
