# Supplementary material for: Prevalence of falls, injury from falls and associations with chronic diseases among community-dwelling older adults in Guangzhou, China: a cross-sectional study
Source: Front Public Health. 2023 Sep 15;11:1251858. doi: 10.3389/fpubh.2023.1251858 (PMC10540617; doi:10.3389/fpubh.2023.1251858)
Supplement: Supplementary file 1 [file Table_1.pdf]

Supplementary material

Supplementary material 1: Questionnaire of health status of elderly people

Part one: Basic information

A1 Your age:

______ years old

A2 Your gender:

①Male ② Female

A3 Your educational level:

①No school ②Primary school

③Secondary school ④College and above

A4 Your marital status:

①Single (unmarried, divorced or widowed) ②Married

A5 Your occupations:

①Worker ②Technical personnel

③Unemployed ④Others

A6 Are you a registered residence in Guangzhou?

①Yes ②No

A7 Your monthly household income per head (¥，RMB):

①≤2000 ②2001-4000

③4001-6000 ④>6000

A8 Do you have medical insurance?

①Yes ②No

Part two: Health status

B1 Have you fallen in the past year? （If you choose "**2**", skip to question "**B3**".）

①Yes（Total of _____ times） ②No

B2 Have you been injured from falls in the past year?

①yes（Total of _____ times） ②No

B3 Have you been diagnosed with any of the following chronic diseases?

None	Yes☐	No☐
Hypertension	Yes☐	No☐
Diabetes	Yes☐	No☐
Coronary heart disease	Yes☐	No☐
Chronic respiratory disease	Yes☐	No☐
Stroke	Yes☐	No☐
Renal disease	Yes☐	No☐
Hyperlipidemia	Yes☐	No☐
Hepatic disease	Yes☐	No☐
High uric acid	Yes☐	No☐
Tumor	Yes☐	No☐
Other chronic disease	Yes☐____________	No☐

B4 Have you felt unwell or had an acute illness in the last 2 weeks?

①Yes ②No

B5 Total of ______ times, you went to the community health service centers for medical treatment in the past year.

B6 How do you feel about your health status, compared to your contemporaries?

①Better than contemporary

②Same as contemporary

③Worse than contemporary

B7 During the last week, how many days did you engage in high intensity physical activities for 10 minutes or more? Such as lifting heavy objects, playing basketball.

______ days per week ☐ No related activities

B8 During the last week, how many days did you engage in moderate intensity physical activity for 10 minutes or more? Such as lifting light objects, playing Tai Chi.

______ days per week ☐ No related activities

B9 During the last week, how many days did you engage in low intensity physical activity 10 minutes or more? Such as taking a walk.

______ days per week ☐ No related activities

B10 During the last week, how long have you been sitting on average every day?

______ hours ____ minutes per day

Supplementary material: Table S1: Comparison of the study population and the seventh national population census of Guangzhou in 2020.

Table S1. Comparison of the study population and the seventh national population census of Guangzhou in 2020.

Variables	Study population, % (n =1629)	Seventh national population census, % (n =1,460,333) [a]	P^b
**Age (%)**			<0.01
65-69	41.80	39.44	
70-74	31.00	24.58	
75-79	17.19	14.74	
≥80-84	10.01	21.24	
**Gender (%)**			<0.01
Male	37.69	46.16	
Female	62.31	53.84	

[a] The categories are grouped according to the Seventh National Population Census of Guangzhou: National Bureau of Statistics of China., 2020.

[b] Differences were found between means within each variable for Chi-square test analysis. And weighted estimates of prevalence of with proportional to population size and post-stratification sample weights adjustment was adopt in this study.
